# Investigating the gene expression profiles of rehabilitated Florida manatees (*Trichechus manatus latirostris*) following red tide exposure

**DOI:** 10.1371/journal.pone.0234150

**Published:** 2020-07-02

**Authors:** Rebecca Lazensky, Margaret E. Hunter, David Moraga Amador, Basima Al-Khedery, Fahong Yu, Cathy Walsh, Matthew A. Gitzendanner, Katie Tripp, Michael T. Walsh, Nancy D. Denslow

**Affiliations:** 1 Department of Physiological Sciences and Center for Environmental & Human Toxicology, University of Florida, Gainesville, Florida, United States of America; 2 Aquatic Animal Health Program, College of Veterinary Medicine, University of Florida, Gainesville, Florida, United States of America; 3 Wetland and Aquatic Research Center, U. S. Geological Survey, Sirenia Project, Gainesville, Florida, United States of America; 4 Interdisciplinary Center for Biotechnology Research, Gainesville, Florida, United States of America; 5 Mote Marine Laboratory, Sarasota, Florida, United States of America; 6 Department of Biology, University of Florida, Gainesville, Florida, United States of America; 7 Florida Museum of Natural History, University of Florida, Gainesville, Florida, United States of America; 8 Save the Manatee Club, Maitland, Florida, United States of America; 9 Large Animal Clinical Sciences, College of Veterinary Medicine, University of Florida, Gainesville, Florida, United States of America; Chang Gung University, TAIWAN

## Abstract

To investigate a Florida manatee (*Trichechus manatus latirostris*) mortality event following a red tide bloom in Southwest Florida, an RNA sequencing experiment was conducted. Gene expression changes in white blood cells were assessed in manatees rescued from a red tide affected area (n = 4) and a control group (n = 7) using RNA sequencing. The genes with the largest fold changes were compared between the two groups to identify molecular pathways related to cellular and disease processes. In total, 591 genes (false discovery rate <0.05) were differentially expressed in the red tide group. Of these, 158 were upregulated and 433 were downregulated. This suggests major changes in white blood cell composition following an exposure to red tide. The most highly upregulated gene, Osteoclast associated 2C immunoglobulin-like receptor (OSCAR), was upregulated 12-fold. This gene is involved in initiating the immune response and maintaining a role in adaptive and innate immunity. The most highly downregulated gene, Piccolo presynaptic cytomatrix protein (PCLO), was downregulated by a factor of 977-fold. This gene is associated with cognitive functioning and neurotransmitter release. Downregulation of this gene in other studies was associated with neuronal loss and neuron synapse dysfunction. Among the cellular pathways that were most affected, immune response, including inflammation, wounds and injuries, cell proliferation, and apoptosis were the most predominant. The pathway with the most differentially expressed genes was the immune response pathway with 98 genes involved, many of them downregulated. Assessing the changes in gene expression associated with red tide exposure enhances our understanding of manatee immune response to the red tide toxins and will aid in the development of red tide biomarkers.

## Introduction

In 2013, a record number of Florida manatee (*Trichechus manatus latirostris*) mortalities (n = 830) were reported, with one-third of those (n = 276) attributed to prior exposure to an expansive and prolonged red tide bloom. Historically, the majority of red tide blooms in Florida occurred in the southwest part of the state with Sarasota serving as the epicenter [1, 2]. This bloom was centered at the mouth of the Caloosahatchee River in Southwest Florida, just south of this epicenter [3]. Florida red tides result when the naturally occurring dinoflagellate species, *Karenia brevis (K*. *brevis)*, ‘blooms’ and emits algal neurotoxins, which have been responsible for numerous mortality episodes of manatees and other wildlife in the past. In free-ranging manatees rescued from red tide conditions, decreased lymphocyte proliferation responses have been observed [4, 5]. In addition, red tide affected manatees have increased superoxide dismutase (SOD) activity, a marker of oxidative stress, in their plasma [4].

The West Indian manatee (*Trichechus manatus)* and the Florida subspecies (*Trichechus manatus latirostris*) are listed as threatened by the Endangered Species Act and the state of Florida [6]. Florida manatee mortalities are primarily due to watercraft strikes, perinatal deaths, and natural causes, including red tide. Historically, several large-scale marine mammal mortality episodes have occurred following red tide blooms including a die-off of 149 manatees in 1996 and an episode involving manatees and dolphins in the early 2000s [7, 8]. When the red tide causing dinoflagellate, *K*. *brevis*, undergoes a rapid population growth under favorable environmental conditions, red tides can form quickly and begin releasing toxins termed “brevetoxins” into the environment in higher concentrations. The brevetoxins released during red tide blooms can have large consequences to fish, seabirds, marine mammals, and humans [8]. Previous studies have shown changes in nerve function, immunotoxicity, signs of oxidative stress, and decreased respiratory function following brevetoxin exposure [8]. Manatees suffer ill health effects following toxin inhalation and consumption of brevetoxin-laced food [9]. Since they are frequently exposed to algal toxins in their natural environments, they serve as an excellent sentinel species for studying how these toxins could potentially impact human health.

To understand the impact of red tide toxins on manatee gene expression, an RNA sequencing (RNA-Seq) experiment was conducted. Differential gene expression of message RNA (mRNA) was compared in manatees rescued from red tide exposure and a non-exposed control group from geographically separated areas, not impacted by red tide (Crystal River, FL and Brevard County, FL). The mRNA transcripts in an organism comprise the transcriptome or the collection of all transcribed RNAs and these can be used to determine an organism’s health status at any given time [10]. Using this technology, we quantified transcript sequences for genes expressed in the white blood cells of normal and red tide impacted manatees. The unique gene expression profiles identified through this investigation may be used in the development of diagnostic assays based on biomarker identification, which could be queried during future unknown mortality episodes. This could help improve diagnostic capacity, rehabilitation efforts, and treatment protocols and better direct future health investigations of unknown mortality episodes.

## Methods

### Manatee buffy coat collection

Manatee white blood cell ‘buffy coat’ samples were opportunistically obtained from two groups (red tide and control) and analyzed using RNA-Seq to detect changes in gene expression (Table 1). The red tide group was comprised of manatees (n = 4) exposed to a prolonged red tide bloom in Lee County, Florida. Blood samples were collected between November 2012 and March 2013 from rescued/stranded manatees that were receiving treatment for red tide-related illnesses at ZooTampa (Tampa, FL) during the peak of the red tide bloom. Two of the manatees were sampled as they came in for treatment (RSW1307 (16) and RSW1310 (17)) and two had been in treatment for a week before they were sampled (103030 (13) and 103054 (14)). Buffy coats were extracted from these samples, frozen immediately at minus 80 °C, and archived at Mote Marine Laboratory in Sarasota, FL. The control group consisted of seven manatees sampled in three locations not impacted by the red tide event. One blood sample was collected in June 2014 from a manatee recovering from pneumonia at ZooTampa, well after the red tide bloom’s resolution. Four samples were collected during the December 2013 manatee health assessments in Crystal River and two were collected in 2013 during the Brevard County health assessments conducted by the U.S. Geological Survey (USGS) Sirenia Project. The current project was approved by several ethics committees including USGS (USGS permit #: MA791721), Florida Fish and Wildlife Conservation Commission (FWC) (FWC permit #: MA067116-1), Federal Fish and Wildlife permit #: MA067116-2 and University of Florida IACUC #201308202. From each animal, peripheral blood samples were collected from the brachial vascular bundle into Lithium-heparin tubes. Samples were centrifuged at ~10–15,000 xg for 10 minutes in an outdoor field setting. White blood cells at the interface between the pelleted red blood cells and the plasma were collected and transferred to RNA*later* (Life Technologies^™^, Carlsbad, CA, USA) immediately in the field, to preserve the buffy coat samples in a ratio of 1.2 mL RNA*later* to <0.5 mL buffy coat according to the manufacturer’s protocol (Life Technologies^™^) and samples were refrigerated at minus 20°C, as recommended by the manufacturer.

**Table 1:** Identification of Florida manatees used in the RNA-Seq study.

Group Name	Same Collection Site	Collection Date	Manatee Identifier/Study ID	Gender	Serum amyloid A (SAA)^a^	Health Notes
					(mg/L)	
Red Tide	ZooTampa	3/13/2013	RSW1307 (16)	M	-	Brevetoxin 4 ng/ml
		2/28/2013	RSW1310 (17)	M	-	Brevetoxin, not measured
		11/6/2012	103030 (13)	M	-	Brevetoxin 9 ng/ml
		2/1/2013	103054 (14)	F	-	Brevetoxin 4 ng/ml
Control	Crystal River, FL	12/4/2013	CCR13-14 (1)	M	18.96	Excellent condition
		12/4/2013	CCR13-19 (7)	M	25.18	Excellent condition
		12/3/2013	CCR13-10 (10)	M	30.63	Excellent condition
		12/4/2013	CCR-13-21(11)	M	17.72	Excellent condition
	Brevard County, FL	12/10/2013	CBC-13-05 (9)	M	16.77	Excellent condition
		12/10/2013	CBC13-02 (12)	M	73.98	Excellent/High SAA
	ZooTampa	6/21/2014	103150 (8)	F	-	Pneumonia case

^a^, SAA values were considered high if they were out of the ‘normal’ range of 0–50 mg/L; SAA values were not collected for manatees at ZooTampa.

Prescreening was done to ensure control manatees did not have any indication of a preexisting illness or trauma, such as cold stress or boat-related injuries. Clinical data from the manatee health assessments were obtained and reviewed to prescreen manatees for inclusion in this study based on blood chemistries and acute phase protein serum amyloid A (SAA) values. SAA measurements were performed on manatee serum by the University of Miami Avian & Wildlife Laboratory (Miami, Florida) following their published protocol [11]. Research has shown that the SAA value is the most reliable indicator of inflammation or illness in manatees [12]. The SAA values for the red tide group and the control manatee with pneumonia were not available but the values from the manatees evaluated during the annual Crystal River (control group) and Brevard manatee health assessments were (Table 1). The control samples were included in the study due to their medically determined ‘healthy’ status, which was based on their healthy blood chemistries, low SAA values (≤50 mg/L; except for one sample), and field examinations during health assessments [12]. The SAA value was only high (74 mg/L) in one manatee (CBC 1302) sampled in Brevard. A complete list of the manatees and associated data used in the study can be found in Table 1 and S1 Table. Samples 14 (red tide) and 8 (control) were female, while the others were male.

### ELISA assay for brevetoxin

Plasma was separated from whole blood via centrifugation at 500 x g, 15 min. Brevetoxin was measured in plasma using modifications of a competitive enzyme linked immunosorbent assay (ELISA) [13] previously used to measure brevetoxins in plasma of manatees [4]. Brevetoxin ELISA kits were purchased from MarbioNC (Wilmington, NC). Plates were coated with BSA-linked PbTx-3 and samples and standards added in serial dilution, with a minimum of seven dilutions and a blank for each. Dilutions were made using PGT (PBS, 0.1% Tween, 0.5% gelatin). Diluted samples and PbTx-3 reference standards were added in 100 μl volume to each well. Goat anti-PbTx-3 was added in 100 μL volume and plates were incubated at room temperature for 1 h using an orbital shaker. After several washes in PBS-T (PBS, 0.1% Tween), a secondary antibody, horse-radish peroxidase-linked rabbit anti-goat IgG, was added and incubated for 1 h at room temperature. After washing again with PBS-T, TMB (3,3’,5,5’-tetramethylbenzidine) substrate was added. Reactions were stopped by adding 100 μL 1.5 M sulfuric acid. Absorbance was read at 450 nm using a microplate reader (BioTek, ELx800, Winooski, VT). Concentrations of brevetoxins in plasma were determined using a standard curve, and results are reported in PbTx-3 equivalents. The limit of detection was approximately 1–2 ng/ml of plasma.

### RNA extraction and purification

RNA was extracted from manatee buffy coat samples according to the Tel-Test Inc. method for total RNA extraction using Stat-60 (Tel-Test, Inc., Friendswood, TX). The purity of the samples was determined using a NanoDrop^™^ 1000 (Thermo Fisher Scientific, Waltham, Massachusetts, USA) to assess 260/280 and 260/230 ratios. To remove DNA contamination, the samples were DN*ase* treated using the TURBO^™^ DNA-*free*^™^ kit according to the manufacturer’s protocol (Life Technologies^™^). After DN*ase* treatment, Qiagen RNeasy columns were used to further purify the samples (Qiagen, Hilden, Germany). The Agilent 2100 Bioanalyzer (Agilent Technologies, Santa Clara, CA, USA) was used to obtain RNA integrity (RIN) values. Most RIN values ranged from 7.3–9.4, with the exception of one that had a RIN value of 5.9 (S1 Table). RNA-Seq was conducted on samples with RIN values >5.9.

### RNA-Seq library preparation and sequencing

The Illumina^®^ EpiCentre ScriptSeq^™^ Complete Gold (Blood)-Low Input Kit (Illumina^®^ EpiCentre, Madison, WI, USA) was used along with the Illumina^®^ FailSafe^™^ PCR Enzyme Mix and ScriptSeq^™^ Index PCR primers (set 1) was used for RNA processing and sequencing library preparation. This kit’s ‘Globin and Ribo-Zero’ reagents were designed to remove globin and rRNA from RNA samples (EpiCentre ScriptSeq^™^, Madison, WI). The Zymo Research RNA Clean and Concentrator-5 (Irvine, CA, USA) was used to purify the RNA (>200 nt) and concentrate the depleted samples. Individual libraries were made for each of the samples which were then pooled at equimolar concentrations. The library pool was prepared for sequencing following the manufacturer’s protocol. The Illumina^®^ NextSeq^®^ 500 (Illumina^®^, San Diego, CA) sequencing platform was used to create paired-end reads using 2 X 150 cycles.

### Bioinformatics and gene expression analysis

Several quality measures were performed using the FastQC [14] on Galaxy, a web-based platform [15] to check the read quality and any potential errors introduced during sequencing or library preparation [16]. The Cutadapt software program [17] was used to remove low quality reads from the final dataset. Reads with a Phred-like score of <40 and bases with a score <20 were excluded. Quality control results can be found in S2 Table. A reference-based approach to alignment was used to compare the sequenced transcripts to the reference manatee genome using the Bowtie2 mapper (v. 2.2.3, [18]). The sequencing of the Florida manatee genome was completed as part of the Broad Institute’s ALLPATHS-LG assembly project using an Illumina HiSeq^™^ instrument and a paired-end whole-genome shotgun approach with partial annotation of the manatee genome [19, 20]. For the alignment, the Broad Institute’s TriManLat1.0 manatee genome assembly (NCBI Accession AHIN00000000) was used as the reference genome [19, 20]. The Bowtie2 mapper [21] was used to independently map the cleaned reads to the reference genome sequences with a “3 mismatches a read” allowance. Proprietary scripts from the Interdisciplinary Center for biotechnology Research (ICBR) at the University of Florida were used along with computational applications SAMtools and the DESeq, EdgeR, [22] in DEB [23] to generate the gene expression data, including fold changes and p-values, to remove duplicates or artifacts from PCR, and to choose uniquely mapped reads for gene expression analysis [24]. Digital gene expression was assessed by counting the number of transcripts that mapped to a gene sequence [23]. For all pairwise comparisons, the DESeq algorithm used a 5% False Discovery Rate (FDR) cutoff [23]. Significant up- and downregulated genes were selected using the FDR adjusted p-value, fold-change, or both for downstream analysis. Both the raw data and RNA-seq data were deposited at NCBI with GEO # GSE86792. Heat maps were generated using GENE-E software [25] with the Linked heat map application using their built in algorithm to display the hierarchical clustering pattern of differential gene expression.

### PathwayStudio^™^ analysis

PathwayStudio^™^ V9 (operating with the ResNet 9.0 database) was used to identify molecular pathways associated with exposure to red tide. The differentially expressed genes were further analyzed by subnetwork enrichment analysis (SNEA) to determine the cell processes involved [26–28]. The analysis involved identifying genes whose protein products work together in pathways. This normally consists of a central regulator and the downstream genes that are coordinately regulated, with pathways based on relationships extracted from the literature. The top 20 pathways related to cell processes were identified.

## Results

### Gene expression results

The RNA-seq samples included in the downstream analysis (n = 11) had a mean number of 57 million reads per sample with a range of 44–70 million reads. The average assembled sequences across samples were 21,420 (range: 19,470 to 25,826). The range in GC content was 40–44% with a mean GC content of 41%. After removal of redundant identifications, a total of 15,301 unique genes were identified by RNA-Seq. Of these, 15,260 mapped to human homologs with E score values <E-10, indicating good confidence in the annotation. A total of 636 genes had 100% match with human homologs and 14,575 had more than 50% identity with human homologs.

**Fig 1:**
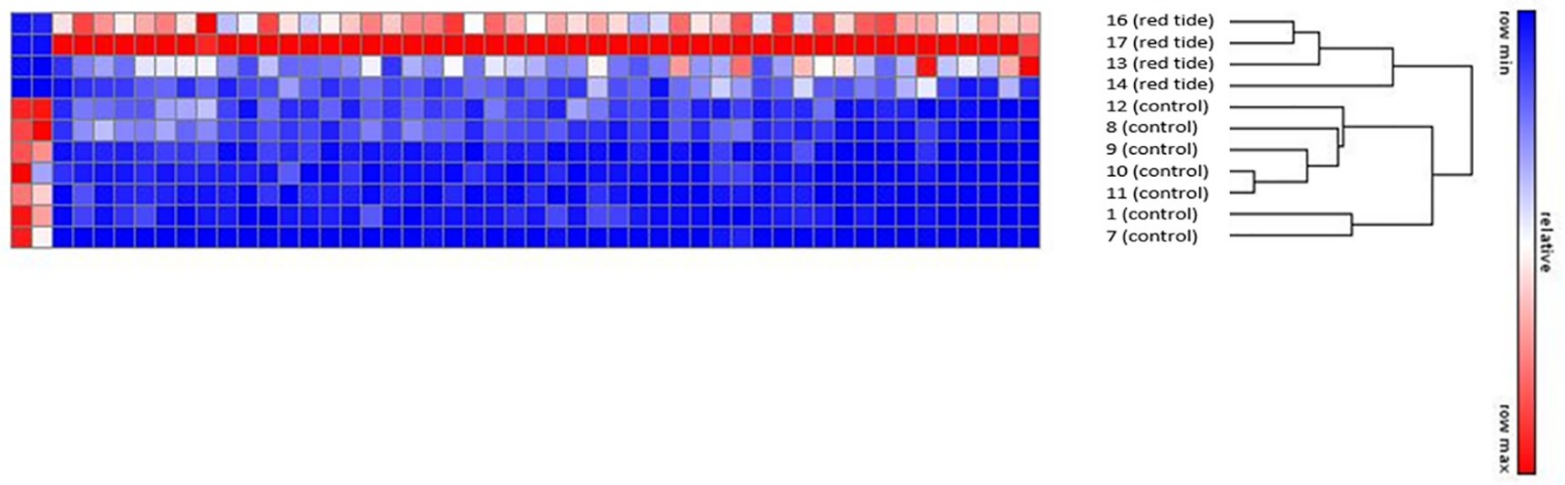
Heat map and dendrogram of individual gene expression data obtained by RNA-Seq for manatees exposed to red tide and an experimental control group. Hierarchical clustering was performed on genes that were differentially expressed (p-value <0.05 and FDR <0.2) and self-grouped based on the clustering pattern as shown by the dendrogram. Red indicates up-regulation and blue indicates down-regulation of gene expression. The intensity of the color is proportional to the degree of up or down-regulation. The sample numbers and red tide or control classification are indicated to the right. Samples 8 and 14 are female; other samples are male.

In Fig 1, the heat map analysis groups the four manatees affected by red tide separately from the “control” manatees. Females clustered with their respective groups and not separately, but with so few females in the sample, it is impossible to know if there is an effect of sex (Fig 1). The animal that was recovering from pneumonia (sample 8) grouped very well with the other control animals, suggesting that it had an RNA profile similar to controls at the time of sampling. The two outliers in the control group, samples 1 and 7 (Fig 1), have expression patterns that are very similar to the rest of the control group and these two manatees were sampled as part of routine health assessments at Crystal River.

The RNA-seq data identified 2,149 genes that were differentially expressed with a p-value <0.05. Considering only adjusted p-values (FDR<0.05), there were 595 differentially expressed genes. The top 50 upregulated and downregulated genes with adjusted p-values are reported in S2 and S4 Tables. Overall, 50 upregulated genes were identified with a log(2) fold change ≥2 (i.e., fold change >4) in the red tide group in comparison to the controls. In the downregulated group, the best 50 genes were significantly downregulated with a log(2) fold change ≤ -5.96 (fold change >62-fold), suggesting that down regulation of gene expression was more pronounced than up regulation.

The 10 upregulated genes with the highest fold change were Osteoclast associated 2C immunoglobulin-like receptor (OSCAR), myotubularin related protein 2 (MTMR2), Transmembrane protein 56 (TMEM56), thymocyte selection associated family member 2 (THEMIS2), matrix metallopeptidase 9 (MMP9), Haptoglobin (HP), Chemokine (C-X-C motif) receptor 2 (CXCR2), Chemokine (C-X-C motif) receptor 1 (CXCR1), monoacylglycerol 0-acyltransferase 2 (MOGAT2) and basic leucine zipper transcription factor 2C ATF-like (BATF) (Table 2). Eight of these are known to be involved in immune system regulation and two in lipid metabolism, which may be linked indirectly to the immune system. The ten downregulated genes with the maximum fold change are piccolo presynaptic cytomatrix protein (PCLO), Interleukin 6 (IL6), zinc finger protein 804B (ZNF804B), family with sequence similarity 1862C member A(FAM186A), ankryn 2C neuronal (ANK2), multimerin 1 (MMRN1), XK 2C Kell blood group complex subunit related family 2C member 6 (XKR6), cysteine-rich 2C angiogenic inducer 2C61 (CYR61), forkhead box A1 (FOXA1), mucin 17 2C cell surface associated (MUC17) (Table 3). Among these genes, two are involved in brain signaling, two are involved as transcriptional factors, and four are involved in the immune system.

**Table 2:** Top 10 upregulated genes of Florida manatees exposed to red tide as compared to the experimental control group.

Gene definition	Gene-symbol	e-value	Fold change	p-value	padj^a^
Osteoclast associated 2C immunoglobulin-like receptor	OSCAR	2.9E-133	12.82	2.0E-03	4.9E-02
Myotubularin related protein 2	MTMR2	0.0E+00	11.59	1.5E-05	1.5E-03
Transmembrane protein 56	TMEM56	1.0E-102	11.55	1.4E-07	3.6E-05
Thymocyte selection associated family member 2	THEMIS2	0.0E+00	10.41	4.1E-06	5.3E-04
Matrix metallopeptidase 9	MMP9	0.0E+00	9.97	1.8E-03	4.5E-02
Haptoglobin	HP	0.0E+00	9.73	1.3E-05	1.3E-03
Chemokine (C-X-C motif) receptor 2	CXCR2	0.0E+00	9.15	1.8E-08	5.6E-06
Chemokine (C-X-C motif) receptor 1	CXCR1	8.0E-174	8.71	7.5E-04	2.6E-02
Monoacylglycerol O-acyltransferase 2	MOGAT2	0.0E+00	8.07	3.3E-06	4.5E-04
Basic leucine zipper transcription factor 2C ATF-like	BATF	6.0E-64	7.70	3.2E-06	4.5E-04

^a^ p value adjusted for false discovery rate.

**Table 3:** Top 10 downregulated genes of Florida manatees exposed to red tide as compared to the experimental control group.

**Gene definition**	**Gene-symbol**	**e-value**	**Fold change**	**p-value**	**padj**^a^
Piccolo presynaptic cytomatrix protein	PCLO	0.0E+00	976.64	7.7E-06	8.7E-04
Interleukin 6	IL6	4.3E-82	635.18	7.0E-09	2.6E-06
Zinc finger protein 804B	ZNF804B	0.0E+00	476.32	3.7E-04	1.6E-02
Family with sequence similarity 186 2C member A	FAM186A	0.0E+00	443.25	3.0E-04	1.4E-02
Ankyrin 2%2C neuronal	ANK2	0.0E+00	403.51	2.5E-05	2.3E-03
Multimerin 1	MMRN1	0.0E+00	353.93	8.4E-04	2.7E-02
XK 2C Kell blood group complex subunit-related family 2C member 6	XKR6	0.0E+00	284.04	1.3E-03	3.6E-02
Cysteine-rich 2C angiogenic inducer 2C 61	CYR61	0.0E+00	273.58	1.1E-03	3.2E-02
Forkhead box A1	FOXA1	0.0E+00	258.22	1.4E-03	3.8E-02
Mucin 17 2C cell surface associated	MUC17	1.1E-118	244.01	1.3E-03	3.5E-02

^a^ p value adjusted for false discovery rate.

### Pathway analysis of cellular and disease processes

**Fig 2:**
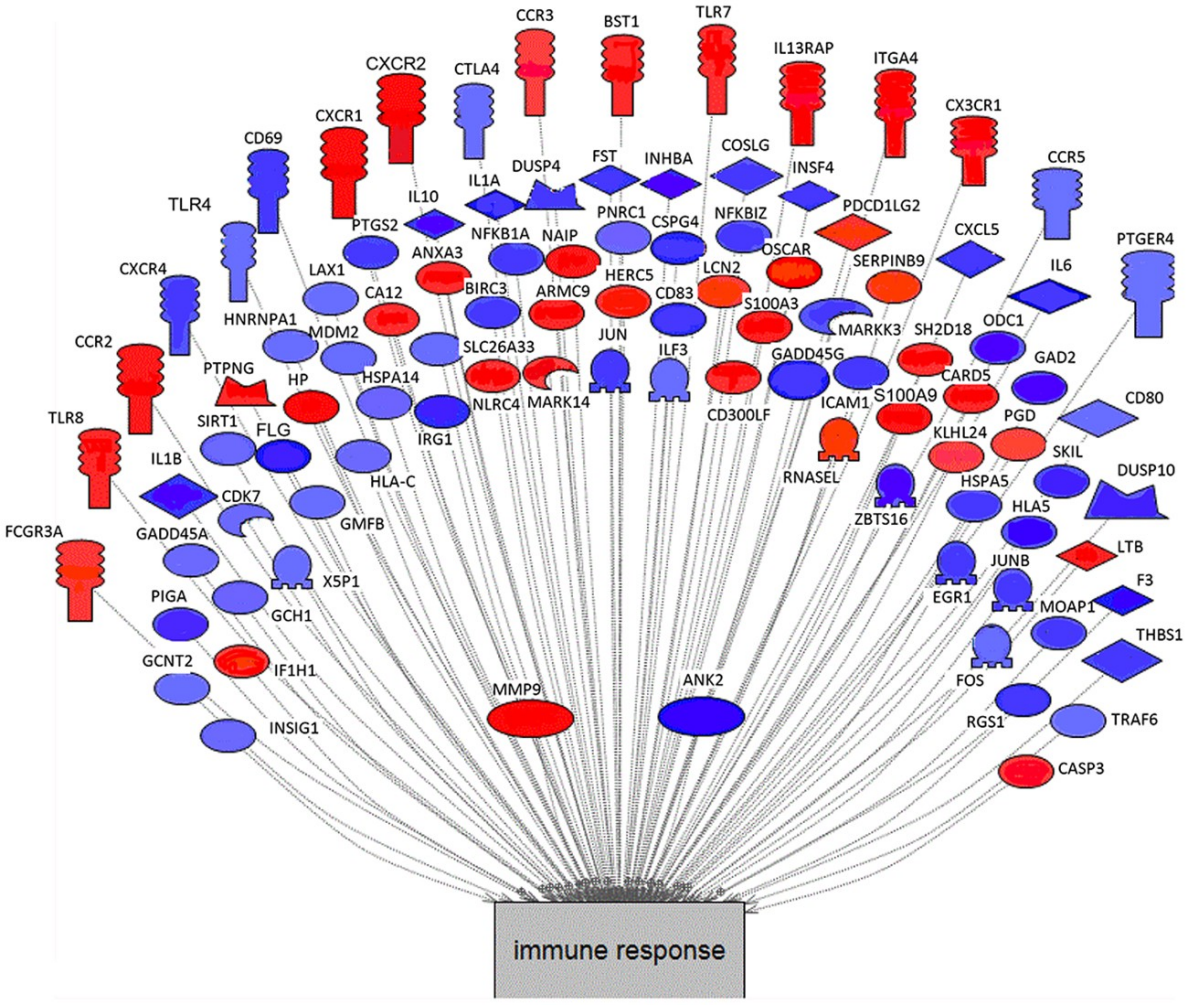
Subnetwork enrichment analysis performed using the software program PathwayStudio^™^ illustrating genes that were altered in white blood cells in relation to immune response in manatees affected by red tide compared to control manatees. Red indicates up-regulation and blue indicates down-regulation of gene expression. The intensity of the color is proportional to the degree of up or down-regulation. See S2 File for symbol definitions.

After comparing the red tide to the control group, the top 595 differentially expressed genes (both up and downregulated) were grouped into pathways involved in cellular and disease processes using PathwayStudio^™^. This program identifies relationships between genes and disease based on knowledge harvested from millions of published articles in PubMed and compares the genes that are differentially expressed in an experiment to the knowledgebase using Fisher’s exact test to derive a statistical inference. The cellular pathway with the most differentially expressed genes (DEGs) was the immune response pathway with 98 genes involved (p-value 3.8 X 10^−17^; Table 4; Fig 2). Many of the genes were downregulated (denoted in blue; Fig 2), suggesting immune suppression. Other cellular pathways of high significance in the top five included cell growth, proliferation, cycle and differentiation (Table 4). Additional cellular pathways related to the immune system that were classified as most affected included cytokine production, inflammatory response, immunity, leukocyte migration, macrophage differentiation, response to stress, innate immune response, and T-cell activation. Pathway Studio also allows one to query pathways related to disease processes (Table 5). The top pathway in this query was neoplasms, followed by inflammation in the 2^nd^ position, and wounds and injuries in the 4^th^ position. Interestingly, colitis was in the 20^th^ position in the list, and some of the manatees present with this ailment (M. Walsh, personal communication).

**Table 4:** Cellular process pathway analysis of genes differentially expressed in red tide and control Florida manatees. The number of overlapping genes between the experimental groups, percentage overlap, and significance of overlap (p-value) are given.

#	Gene Set Seed	Total # of Genes^a^	Overlapping Genes^b^	Percent Overlap (%)	p-value
1	Immune response	1791	98	5	3.57E-17
2	Cell growth	3729	155	4	1.74E-16
3	Cell proliferation	5831	207	3	1.51E-15
4	Cell cycle	3237	138	4	3.09E-15
5	Cell differentiation	5167	185	3	1.33E-13
6	Apoptosis	5546	194	3	1.89E-13
7	Cytokine production	1034	60	5	1.43E-11
8	Osteoclast formation	304	29	9	7.24E-11
9	Inflammatory response	1404	70	4	2.22E-10
10	Osteoclast differentiation	328	29	8	4.40E-10
11	DNA replication	1823	80	4	3.81E-09
12	Cell death	3381	124	3	4.96E-09
13	Immunity	1239	61	4	6.40E-09
14	Leukocyte migration	541	36	6	7.93E-09
15	Translation	1382	65	4	1.12E-08
16	Macrophage differentiation	210	21	9	1.44E-08
17	Response to stress	317	26	8	1.73E-08
18	Innate immune response	458	32	6	1.80E-08
19	T-cell activation	1018	52	5	3.02E-08
20	Vascularization	1958	81	4	4.22E-08

^a^ The total number of genes that belong to a particular pathway based on scanning pubmed.

^b^ The number of genes that are significantly altered in the red tide animals in the particular pathway.

**Table 5:** Disease pathway analysis of differentially expressed genes in red tide and control Florida manatees selected by subnetwork enrichment. The number of overlapping genes between the two groups, percentage overlap, and significance of overlap (p-value) are given.

#	Gene Set Seed	Total # of Genes	Overlapping Genes	Percent Overlap (%)	p-value
1	Neoplasms	4697	157	3	4.62E-16
2	Inflammation	2351	99	4	2.88E-15
3	Cancer	3014	111	3	3.35E-13
4	Wounds and Injuries	2035	85	4	9.07E-13
5	Ischemia	993	54	5	2.20E-12
6	Leukemia	821	48	5	3.48E-12
7	Arthritis, Rheumatoid	665	42	6	8.04E-12
8	Neoplasm Metastasis	1947	80	4	1.25E-11
9	Infection	1914	78	4	3.69E-11
10	Liver Neoplasms	818	46	5	3.90E-11
11	Asthma	787	44	5	1.32E-10
12	Brain Ischemia	532	35	6	1.74E-10
13	Arthritis	657	39	5	2.99E-10
14	Stomach Neoplasms	783	43	5	3.83E-10
15	Atherosclerosis	1179	55	4	4.72E-10
16	Lymphoma	497	33	6	4.75E-10
17	Lung Neoplasms	1060	51	4	7.86E-10
18	Intimal hyperplasia	332	26	7	1.21E-09
19	Skin Neoplasms	358	27	7	1.31E-09
20	Colitis	606	36	5	1.51E-09

^a^ The total number of genes that belong to a particular pathway based on scanning pubmed.

^b^ The number of genes that are significantly altered in the red tide animals in the particular pathway.

## Discussion

Brevetoxins are neurotoxins that primarily affect voltage-gated sodium channels in nerve cells [29]. While they can disrupt neurological processes, brevetoxins also can cause systemic organ failure in the liver and kidney in manatees and humans [7, 30]. There is also a primary response from the immune system to brevetoxins, with alterations in alveolar macrophages and in the brain, changes in lymphocytes and microglial cells [4]. The release of inflammatory mediators and initiation of apoptosis have also been observed, with these conditions leading to death [7].

While the manatee genome sequence is not completely annotated, we used it as a scaffold for Illumina reads and then annotated the genes by BLAST against the human genome. The analysis provided excellent annotation by comparing sequences with human homologs. This great similarity between manatee and human transcriptomes allowed us to use pathway analysis to identify pathways most likely affected by the exposures. Because manatees are mammals, it is reasonable to assume that many of the basic pathways described for other mammals likely operate in the same way in manatees; although manatees probably utilize species-specific pathways as well. Transcriptomics analysis of white blood cells in the current study provided molecular data to support previous gross pathology of red tide-affected manatees. Bossart et al [7] found that manatees that died from exposure to red tide toxins had multiorgan congestion, hemorrhage and edema, with the lung primarily affected. They also found large amounts of blood and other fluids oozing from cut surfaces. Microscopic examination by Bossart et al. showed brevetoxin in the macrophages and lymphocytes and general inflammation that may have resulted in a cytokine cascade of events leading to death. In short, their analysis of “neurointoxication, hemolytic anemia and immunologic compromise” was supported by this molecular data.

Among the cellular pathways that were most affected by brevetoxin exposure, immune response was the predominant pathway with a p-value of 3.6 X 10^−17^ (Table 4 and Fig 2). In the top 20 pathways, eight other pathways were also related to immune function and one was related to vascularization (Table 4). Linking gene expression changes to disease also identified many immune-related diseases including inflammation, wounds and injuries, infection, and colitis (Table 5). Significantly, colitis was identified as one of the gene expression pathways related to disease correlating with one of the clinical findings for manatees that were treated for red tide (M. Walsh, personal communication). Among the disease pathways, there are several gene expression changes relating to neoplasms (rapid growth of tissue, benign or cancerous), although neoplasms have not been documented in manatees killed by red tide. Outside of manatees, studies have linked brevetoxin exposure with DNA damage in Jurkat E6-1 cells [31, 32]. These cells are important multipotent cells in the immune system of humans, giving rise to both Th1 and Th2 type lymphocytes, and could be targets for further investigations for the effects of chronic exposure to red tide in manatees.

An in depth analysis of the immunoglobulin heavy chain locus in the manatee genome has shown that the Florida manatee has a limited number of segmental diversity compared to other mammals [33], raising the question about how well it can defend itself from opportunistic parasites. Breaux et al [33] have shown that the heavy chain locus can give rise to only 3,744 combinations from its 13 V segments, 48 D segments and 6 J segments. This is about half of the human repertoire (with 6,072 possible combinations) and about 14% of the elephant repertoire (possible 26,622 combinations). Yet, the manatee is thought to have a relatively strong immune system [34]. Potentially manatees rely more heavily on cell-mediated immunity, as suggested by Breaux et al [33]. While this question was out of the scope of the current project, it would be interesting to follow up this idea in subsequent experiments.

At the level of the individual gene changes in manatees, we observed changes in gene functions related to immune response (IL6, ZNF804B), leukocyte production and differentiation (MMP9, OSCAR), and neuronal activity and synapse (MTMR2, PCLO, ANK2). The most differentially expressed gene, piccolo presynaptic cytomatrix protein (PCLO), was downregulated (977-fold change; adjusted p-value, 9.0 X 10^−4^). PCLO is associated with neuronal loss and synapse, neurotransmitter release, and cognitive dysfunction [35, 36]. The most upregulated gene, OSCAR (13-fold change; adjusted p-value, 4.9 X 10^−2^), is associated with both immunity and leukocyte activity. Lastly the MMP9 gene, which is associated with leukocyte migration, was upregulated (10-fold change; p-value, 4.5 X 10^−2^). Functional information about each of the ten most up- or downregulated genes in mammals appears in the S1 File. In conclusion, many individual genes known to be involved in mammalian immune response, inflammation, and neurodegenerative processes were altered dramatically in the white blood samples of manatees and these may serve as biomarkers of exposure to red tide.

By examining the biomarker activity in manatees exposed to red tide, we can add to the growing body of research which continues to identify neurological impacts and the potential of DNA damage associated with exposure to harmful algal blooms in both animals and humans [31, 32, 37]. The biomarker activity manatees displayed following algal bloom exposures could be used to inform the epidemiological implications of these aquatic toxins on human health [30]. Future work will be required to develop sensitive and specific diagnostic assays using these biomarker genes, which would be useful for rapid identification of disease in manatees. Biomarker testing could also be used to help investigate unknown single-case manatee deaths [38].

Our control group was sampled during the annual manatee health assessments in Crystal River and Brevard County FL, two areas not impacted by the 2012–2013 red tide event. Because it is possible that manatees from the control group may have visited the area where the red tide occurred, we cannot be entirely certain the manatees in our group were free of any red tide exposure. However, the control manatees were distinct in their mRNA expression patterns compared to the red tide group. Additional limitations to the study were the low number of biological replicates and the inability to sample the tissues that are most targeted by red tide, including the lungs, liver, kidney and brain [7]. We were limited to white blood cells and their changes in gene expression and thus, validation of the potential biomarkers will require more in-depth studies.

Two of the red tide samples (#16 and #17) were collected from manatees within 24 hours of their arrival at ZooTampa for rehabilitation and two (#13 and #14) were collected from manatees after they received a one-week course of treatment for red tide-related illnesses at the Zoo. As expected, the two groups showed minimal separation from each other in the heat map (Fig 1), with the two manatees in early recovery grouping more closely together. The manatees that had received treatment for a week at ZooTampa before they were sampled showed the same differentially regulated genes when compared to manatees that were sampled immediately upon arrival, but the intensity of the change was smaller. All four of the red tide manatees grouped together as a larger group and were distinct from the controls.

## Conclusion

Gene expression changes related to immune response, neurodegenerative processes, and inflammation were the most prominent observations in our study in response to brevetoxin exposure. This aligns with the research findings of other studies which showed that the health effects of red tide exposures are not only gastrointestinal and respiratory, but neurological as well, which is emerging as an increasing concern [4, 37]. Additional research should be done to study the long-term health effects of algal toxin exposures on animals and humans beyond their acute effects. Detecting these and other variations in manatee gene expression profiles can aid researchers in their efforts to further understand the health effects of red tide toxins on manatee health and continually improve manatee rehabilitation practices and the treatment of red tide-related illnesses.

## Supporting information

S1 Table Information about sequenced samples. (DOCX)

S2 Table Quality control results of the RNAseq analysis. (DOCX)

S3 Table Top 50 upregulated genes of Florida manatees exposed to red tide. (DOCX)

S4 Table Top 50 downregulated genes of Florida manatees exposed to red tide. (DOCX)

S1 File Gene function for the ten most upregulated and ten most down-regulated genes in the transcriptomics experiment. (DOCX)

S2 File Symbol definitions for Fig 2. (DOCX)
