# Supplementary material for: Investigating the gene expression profiles of rehabilitated Florida manatees (Trichechus manatus latirostris) following red tide exposure
Source: PLoS One. 2020 Jul 2;15(7):e0234150. doi: 10.1371/journal.pone.0234150 (PMC7331979; doi:10.1371/journal.pone.0234150)
Supplement: S1 Table — (DOCX) [file pone.0234150.s001.docx]

Supplemental Table 1. Information About Sequenced Samples (n=11)

| Sample group | Sample name | Date of collection | Gender | Health notes | RIN^a^ |
| --- | --- | --- | --- | --- | --- |
| Red tide | RSW 1307 (16) | 3/13/2013 | M | Brevetoxin 4 ng/ml | 8.7 |
| Red tide | RSW 1310 (17) | 2/28/2013 | M | No brevetoxin data | 9.4 |
| Red tide | 103030 (13) | 11/6/2012 | M | Brevetoxin 9 ng/ml | 8.6 |
| Red tide | RSW 1302 (103054) (14) | 2/1/2013 | F | Brevetoxin 4 ng/ml | 7.6 |
| Control | CBC-13-05 (9) | 12/10/2013 | M | Excellent condition | 8.2 |
| Control | CBC-13-02 (12) | 12/10/2013 | M | Excellent condition/high SAA | 8.7 |
| Control | 103150 (8) | 6/21/2014 | F | Pneumonia case | 9.3 |
| Control | CCR13-14 (1) | 12/4/2013 | M | Excellent condition | 8.5 |
| Control | CCR13-19 (7) | 12/4/2013 | M | Excellent condition | 7.3 |
| Control | CCR13-10 (10) | 12/3/2013 | M | Excellent condition | 5.9 |
| Control | CCR-13-21 (11) | 12/4/2013 | M | Excellent condition | 8.6 |

^a^RIN, RNA integrity number
