# Supplementary material for: Investigating the gene expression profiles of rehabilitated Florida manatees (Trichechus manatus latirostris) following red tide exposure
Source: PLoS One. 2020 Jul 2;15(7):e0234150. doi: 10.1371/journal.pone.0234150 (PMC7331979; doi:10.1371/journal.pone.0234150)
Supplement: S2 Table — (DOCX) [file pone.0234150.s002.docx]

Supplemental Table 2. Quality Control Results of the RNA-seq analysis

GC%: percentage of guanine-cytosine nucleotides

N % content: ambiguous base calls

K-mers: ‘strings’ or combinations of short stretches of DNA which are overrepresented in the transcriptome.
