# Supplementary material for: Investigating the gene expression profiles of rehabilitated Florida manatees (Trichechus manatus latirostris) following red tide exposure
Source: PLoS One. 2020 Jul 2;15(7):e0234150. doi: 10.1371/journal.pone.0234150 (PMC7331979; doi:10.1371/journal.pone.0234150)
Supplement: S3 Table — (DOCX) [file pone.0234150.s003.docx]

Supplemental Table 3. Top 50 Upregulated Genes of Florida Manatees Exposed to Red Tide

| **Gene-def** | **Gene-symbol** | **e-value** | **Fold change** | **pval** | **padj** |
| --- | --- | --- | --- | --- | --- |
| Osteoclast associated 2C immunoglobulin-like receptor | OSCAR | 2.9E-133 | 12.82 | 2.0E-03 | 4.9E-02 |
| Myotubularin related protein 2 | MTMR2 | 0.0E+00 | 11.59 | 1.5E-05 | 1.5E-03 |
| Transmembrane protein 56 | TMEM56 | 1.0E-102 | 11.55 | 1.4E-07 | 3.6E-05 |
| Thymocyte selection associated family member 2 | THEMIS2 | 0.0E+00 | 10.41 | 4.1E-06 | 5.3E-04 |
| Matrix metallopeptidase 9 | MMP9 | 0.0E+00 | 9.97 | 1.8E-03 | 4.5E-02 |
| Haptoglobin | HP | 0.0E+00 | 9.73 | 1.3E-05 | 1.3E-03 |
| Chemokine (C-X-C motif) receptor 2 | CXCR2 | 0.0E+00 | 9.15 | 1.8E-08 | 5.6E-06 |
| Chemokine (C-X-C motif) receptor 1 | CXCR1 | 8.0E-174 | 8.71 | 7.5E-04 | 2.6E-02 |
| Monoacylglycerol O-acyltransferase 2 | MOGAT2 | 0.0E+00 | 8.07 | 3.3E-06 | 4.5E-04 |
| Basic leucine zipper transcription factor 2C ATF-like | BATF | 6.0E-64 | 7.70 | 3.2E-06 | 4.5E-04 |
| Nuclear factor 2C erythroid 2 | NFE2 | 7.6E-176 | 7.66 | 7.5E-10 | 3.8E-07 |
| Stimulator of chondrogenesis 1 | SCRG1 | 2.4E-57 | 7.26 | 6.1E-04 | 2.3E-02 |
| RAB GTPase activating protein 1-like | RABGAP1L | 2.8E-141 | 7.18 | 5.5E-09 | 2.1E-06 |
| ADP-ribosylation factor-like 11 | ARL11 | 4.3E-101 | 6.95 | 1.9E-08 | 5.9E-06 |
| G protein-coupled receptor 15 | GPR15 | 0.0E+00 | 6.89 | 9.1E-05 | 5.9E-03 |
| Serpin peptidase inhibitor 2C clade B 2C member 11 | SERPINB11 | 0.0E+00 | 6.82 | 8.1E-06 | 9.2E-04 |
| UBX domain protein 8 | UBXN8 | 1.3E-27 | 6.69 | 6.7E-04 | 2.4E-02 |
| S100 calcium binding protein A9 | S100A9 | 1.5E-44 | 6.50 | 6.6E-05 | 4.7E-03 |
| NLR family 2C apoptosis inhibitory protein | NAIP | 0.0E+00 | 6.34 | 3.3E-07 | 6.8E-05 |
| Chemokine (C-C motif) receptor 2 | CCR2 | 0.0E+00 | 6.21 | 2.4E-07 | 5.4E-05 |
| Protein phosphatase 1 2C regulatory subunit 3B | PPP1R3B | 0.0E+00 | 5.84 | 8.4E-08 | 2.3E-05 |
| Solute carrier organic anion transporter family 2C member 4C1 | SLCO4C1 | 0.0E+00 | 5.67 | 9.4E-04 | 2.9E-02 |
| Spindle and kinetochore associated complex subunit 1 | SKA1 | 7.6E-137 | 5.49 | 1.0E-04 | 6.4E-03 |
| Histone cluster 2 2C H4a | HIST2H4A | 4.7E-53 | 5.33 | 7.3E-04 | 2.5E-02 |
| Uridine phosphorylase 1 | UPP1 | 1.7E-169 | 5.31 | 1.1E-06 | 1.8E-04 |
| V-set and transmembrane domain-containing protein 1 | AC012314.28 | 1.7E-37 | 5.23 | 1.8E-05 | 1.8E-03 |
| S100 calcium binding protein A8 | S100A8 | 6.0E-41 | 5.14 | 1.4E-03 | 3.9E-02 |
| Aquaporin 11 | AQP11 | 2.3E-163 | 5.10 | 1.3E-05 | 1.3E-03 |
| Peptidyl arginine deiminase 2C type II | PADI2 | 0.0E+00 | 4.93 | 1.2E-03 | 3.4E-02 |
| Cytochrome P450 2C polypeptide 1 | CYP2R1 | 0.0E+00 | 4.90 | 2.0E-03 | 4.9E-02 |
| SEC14-like 1 (S. cerevisiae) | SEC14L1 | 0.0E+00 | 4.88 | 4.1E-05 | 3.3E-03 |
| Lymphotoxin beta (TNF superfamily 2C member 3) | LTB | 2.3E-121 | 4.81 | 1.1E-04 | 7.0E-03 |
| P450 (cytochrome) oxidoreductase | POR | 0.0E+00 | 4.80 | 3.3E-04 | 1.5E-02 |
| Chromosome 12 open reading frame 4 | C12orf4 | 0.0E+00 | 4.76 | 5.3E-06 | 6.5E-04 |
| Vanin 2 | VNN2 | 0.0E+00 | 4.72 | 4.5E-07 | 8.8E-05 |
| Disrupted in renal carcinoma 2 | DIRC2 | 0.0E+00 | 4.68 | 4.7E-06 | 6.0E-04 |
| Colony stimulating factor 3 receptor (granulocyte) | CSF3R | 0.0E+00 | 4.68 | 2.2E-07 | 5.0E-05 |
| ELOVL fatty acid elongase 3 | ELOVL3 | 4.2E-139 | 4.67 | 2.8E-05 | 2.4E-03 |
| Sphingosine-1-phosphate receptor 4 | S1PR4 | 3.7E-174 | 4.67 | 3.8E-04 | 1.7E-02 |
| Complement component 1 2C r subcomponent-like | C1RL | 0.0E+00 | 4.66 | 5.4E-06 | 6.6E-04 |
| Isocitrate dehydrogenase 1 (NADP+) 2C soluble | IDH1 | 0.0E+00 | 4.64 | 2.7E-07 | 5.7E-05 |
| Caspase recruitment domain family 2C member 6 | CARD6 | 0.0E+00 | 4.40 | 1.4E-04 | 8.2E-03 |
| Armadillo repeat containing 9 | ARMC9 | 0.0E+00 | 4.40 | 5.5E-04 | 2.1E-02 |
| Ninein (GSK3B interacting protein) | NIN | 0.0E+00 | 4.33 | 8.4E-05 | 5.6E-03 |
| Sulfotransferase family 2C member 4 | SULT1C4 | 0.0E+00 | 4.28 | 2.3E-06 | 3.5E-04 |
| Chromosome 7 open reading frame 57 | C7orf57 | 4.8E-157 | 4.27 | 1.0E-04 | 6.4E-03 |
| Toll-like receptor 8 | TLR8 | 0.0E+00 | 4.27 | 1.4E-04 | 8.1E-03 |
| Interferon-induced protein with tetratricopeptide repeats 1 | IFIT1 | 0.0E+00 | 4.26 | 6.5E-04 | 2.4E-02 |
| Leukocyte specific transcript 1 | LST1 | 8.7E-33 | 4.23 | 1.1E-03 | 3.3E-02 |
| Transcription factor Dp-2 (E2F dimerization partner 2) | TFDP2 | 0.0E+00 | 4.17 | 4.5E-05 | 3.5E-03 |
