# Supplementary material for: Investigating the gene expression profiles of rehabilitated Florida manatees (Trichechus manatus latirostris) following red tide exposure
Source: PLoS One. 2020 Jul 2;15(7):e0234150. doi: 10.1371/journal.pone.0234150 (PMC7331979; doi:10.1371/journal.pone.0234150)
Supplement: S4 Table — (DOCX) [file pone.0234150.s004.docx]

Supplemental Table 4. Top 50 Downregulated Genes of Florida Manatees Exposed to Red Tide

| **Gene-def** | **Gene-symbol** | **e-value** | **Fold change** | **pval** | **padj** |
| --- | --- | --- | --- | --- | --- |
| Piccolo presynaptic cytomatrix protein | PCLO | 0.0E+00 | 976.64 | 7.7E-06 | 8.7E-04 |
| Interleukin 6 | IL6 | 4.3E-82 | 635.18 | 7.0E-09 | 2.6E-06 |
| Zinc finger protein 804B | ZNF804B | 0.0E+00 | 476.32 | 3.7E-04 | 1.6E-02 |
| Family with sequence similarity 186 2C member A | FAM186A | 0.0E+00 | 443.25 | 3.0E-04 | 1.4E-02 |
| Ankyrin 2%2C neuronal | ANK2 | 0.0E+00 | 403.51 | 2.5E-05 | 2.3E-03 |
| Multimerin 1 | MMRN1 | 0.0E+00 | 353.93 | 8.4E-04 | 2.7E-02 |
| XK 2C Kell blood group complex subunit-related family 2C member 6 | XKR6 | 0.0E+00 | 284.04 | 1.3E-03 | 3.6E-02 |
| Cysteine-rich 2C angiogenic inducer 2C 61 | CYR61 | 0.0E+00 | 273.58 | 1.1E-03 | 3.2E-02 |
| Forkhead box A1 | FOXA1 | 0.0E+00 | 258.22 | 1.4E-03 | 3.8E-02 |
| Mucin 17 2C cell surface associated | MUC17 | 1.1E-118 | 244.01 | 1.3E-03 | 3.5E-02 |
| Mucin 3A 2C cell surface associated | MUC3A | 3.7E-180 | 228.83 | 5.7E-05 | 4.2E-03 |
| GRP1 -associated scaffold protein | GRASP | 1.8E-164 | 221.90 | 9.9E-10 | 4.8E-07 |
| Matrix metallopeptidase 1 | MMP1 | 0.0E+00 | 207.19 | 1.3E-03 | 3.6E-02 |
| SPATA31 subfamily D 2C member 1 | SPATA31D1 | 0.0E+00 | 205.88 | 7.8E-04 | 2.6E-02 |
| Filaggrin | FLG | 4.0E-107 | 197.32 | 7.4E-05 | 5.1E-03 |
| Adhesion G protein-coupled receptor L3 | ADGRL3 | 0.0E+00 | 192.44 | 1.1E-03 | 3.2E-02 |
| Family with sequence similarity 205 2C member A | FAM205A | 0.0E+00 | 186.28 | 1.8E-03 | 4.5E-02 |
| Chromosome 2 open reading frame 78 | C2orf78 | 0.0E+00 | 185.21 | 8.7E-05 | 5.7E-03 |
| Glutamate receptor 2C metabotropic 8 | GRM8 | 0.0E+00 | 183.47 | 9.0E-04 | 2.8E-02 |
| KIAA2022 | KIAA2022 | 0.0E+00 | 182.46 | 1.3E-03 | 3.6E-02 |
| Kelch-like family member 1 | KLHL1 | 0.0E+00 | 179.69 | 1.3E-03 | 3.6E-02 |
| Interleukin 1 2C alpha | IL1A | 5.3E-84 | 178.79 | 1.8E-07 | 4.4E-05 |
| Unc-79 homolog (C. elegans) | UNC79 | 0.0E+00 | 168.31 | 1.0E-03 | 3.1E-02 |
| Transmembrane protein 132B | TMEM132B | 0.0E+00 | 162.79 | 2.4E-04 | 1.2E-02 |
| WD repeat domain 87 | WDR87 | 0.0E+00 | 157.52 | 2.5E-04 | 1.2E-02 |
| Immunoglobulin superfamily 2C member 10 | IGSF10 | 0.0E+00 | 150.87 | 2.7E-04 | 1.3E-02 |
| semaphorin 3A | SEMA3A | 0.0E+00 | 136.57 | 3.1E-04 | 1.5E-02 |
| Protein tyrosine phosphatase 2C receptor-type 2C Z polypeptide 1 | PTPRZ1 | 0.0E+00 | 120.18 | 1.9E-03 | 4.6E-02 |
| Growth arrest and DNA-damage-inducible 2C gamma | GADD45G | 6.0E-110 | 117.58 | 3.0E-04 | 1.4E-02 |
| Paired box 9 | PAX9 | 0.0E+00 | 117.37 | 1.6E-04 | 8.9E-03 |
| G protein-coupled receptor 158 | GPR158 | 0.0E+00 | 113.61 | 8.7E-05 | 5.7E-03 |
| Coagulation factor III (thromboplastin 2C tissue factor) | F3 | 1.2E-115 | 112.96 | 1.4E-04 | 8.1E-03 |
| Protocadherin beta 5 | PCDHB5 | 0.0E+00 | 108.65 | 7.3E-04 | 2.5E-02 |
| C-C motif chemokine 4 MIP-1-beta(3-69) | AC243829.1 | 2.1E-46 | 107.49 | 1.1E-20 | 3.0E-17 |
| Protein tyrosine phosphatase 2C receptor type 2C B | PTPRB | 0.0E+00 | 104.89 | 3.6E-04 | 1.6E-02 |
| Ret finger protein-like 1 | RFPL1 | 1.1E-105 | 100.35 | 5.3E-04 | 2.1E-02 |
| NACHT and WD repeat domain containing 2 | NWD2 | 0.0E+00 | 95.38 | 4.4E-04 | 1.8E-02 |
| Heparin-binding EGF-like growth factor | HBEGF | 1.3E-89 | 94.91 | 3.1E-12 | 2.5E-09 |
| Zinc finger homeobox 4 | ZFHX4 | 0.0E+00 | 93.19 | 3.9E-04 | 1.7E-02 |
| SPATA31 subfamily 2C member 6 | SPATA31A6 | 8.7E-158 | 87.42 | 9.5E-06 | 1.0E-03 |
| Pappalysin 2 | PAPPA2 | 0.0E+00 | 87.06 | 6.5E-04 | 2.4E-02 |
| Mucin 6 2C oligomeric mucus/gel-forming | MUC6 | 0.0E+00 | 85.47 | 1.0E-07 | 2.7E-05 |
| Protocadherin 11 X-linked | PCDH11X | 0.0E+00 | 81.05 | 1.3E-03 | 3.6E-02 |
| Homeobox D10 | HOXD10 | 0.0E+00 | 80.98 | 1.8E-03 | 4.5E-02 |
| Tripartite motif containing 42 | TRIM42 | 0.0E+00 | 80.56 | 3.5E-04 | 1.6E-02 |
| Usher syndrome 2A (autosomal recessive 2C mild) | USH2A | 0.0E+00 | 77.51 | 1.3E-03 | 3.6E-02 |
| Dachsous cadherin-related 1 | DCHS1 | 0.0E+00 | 76.49 | 1.1E-03 | 3.2E-02 |
| Potassium channel 2C voltage gated subfamily A 2C member 4 | KCNA4 | 0.0E+00 | 63.25 | 2.1E-03 | 5.0E-02 |
| Myosin 2C heavy chain 2 2C skeletal muscle 2C adult | MYH2 | 0.0E+00 | 62.67 | 1.1E-03 | 3.2E-02 |
| Chondroitin sulfate proteoglycan 4 | CSPG4 | 0.0E+00 | 62.21 | 6.1E-04 | 2.3E-02 |
