# Supplementary material for: Investigating the gene expression profiles of rehabilitated Florida manatees (Trichechus manatus latirostris) following red tide exposure
Source: PLoS One. 2020 Jul 2;15(7):e0234150. doi: 10.1371/journal.pone.0234150 (PMC7331979; doi:10.1371/journal.pone.0234150)
Supplement: S1 File — (DOCX) [file pone.0234150.s005.docx]

S1 File. Gene Function for the Ten Most Up-regulated and Ten Most Down-regulated Genes in the Transcriptomics Experiment.

*Up-regulated genes and their functions*

The **osteoclast associated, immunoglobulin-like receptor (OSCAR)** gene (up-regulated by a factor of 12-fold) is a member of the leukocyte receptor complex which performs several functions including bone homeostasis and re-absorption (1). It initiates immune response and maintains a role in adaptive and innate immunity (2).

**Chemokine (C-X-C motif) receptor 2 (CXCR2)** and **Chemokine (C-X-C motif) receptor 1 (CXCR1)** (both up-regulated by a factor of 9-fold), are receptors for IL8, also involved in the immune response (3). These findings suggest that the immune system of manatees exposed to red tide is trying to mount an immune response to red tide toxins.

The **thymocyte selection associated family member 2 (THEMIS2)** gene supports the production of tumor necrosis factor (TNFα) and thymocyte development (4). Themis2 protein acts on macrophages and signals pathway specific effects on toll like receptors (5). Themis2 protein is a known tumor angiogenic factor, which is increased drastically in inflammatory cells and allergic and inflammatory lung disease (6).

**Matrix metallopeptidase 9 (gelatinase B, 92kDa gelatinase, 92kDa type IV collagenase) (MMP9)** is thought to assist extracellular matrix proteolysis and leukocyte migration (7). In addition, the protein encoded by MMP9 degrades fibronectin and is potentially involved in osteoclastic resorption in bone. **Transmembrane protein 56-like (TMEM56)** (up-regulated by a factor of 11-fold) is an integral membrane protein whose upregulation was associated with hypertension and an increased likelihood of having coronary heart disease (CHD) in a previous gene expression study (8).

**Myotubularin related protein 2, transcript variant 2 (MTMR2)** is involved in phosphatase activity (9). MTMR2 is located in motor and sensory neurons and Schwann cells, which are sites where myelin is formed (10). MTMR2 is thought to have a role in enhancing peripheral nerve functioning (9). Changes in these types of genes suggests a direct effect of brevetoxins on the nervous system, which is consistent with the findings of a previous study that showed the brevetoxins bind with high affinity to brain synaptosomes (11).

**The gene stimulator of chondrogenesis 1 (SCRG1)** (up-regulated by 7-fold) is also involved in neurodegenerative changes (12) and may be responding to brevetoxins.

**Complement component 1 2C r subcomponent-like (C1RL),** a gene involved in the complement cascade, innate immune response, and cleavage of prohaptoglobin to its active form in the endoplasmic reticulum (13) was up-regulated by 4.7-fold. **Haptoglobin (HP)** is an acute phase protein (up-regulated 9.7-fold in this study) that supports inflammation and T cell immune response (14). It binds free hemoglobin, prevents iron loss, and performs antibacterial activity (15). While complement cascade and acute phase proteins are typically increased during inflammation, several genes are typically down-regulated during an acute phase response.

**Transthyretin (TTR)** is an acute phase protein encoded in a gene, which is typically down-regulated during immune response and inflammation. These findings were similar to results seen in a separate proteomics survey performed by several of the authors of this study using the same manatee samples where increased acute phase complement cascade proteins C3 and C4-A were also increased while TTR was decreased (16). These results were consistent with other studies of the positive and negative acute phase gene profiles associated with inflammation, immune response, and trauma (17).

*Down-regulated genes and their functions*

**The gene piccolo presynaptic cytomatrix protein (PCLO)** is associated with cognitive functioning and neurotransmitter release (18). In a previous study, PCLO absence was associated with neuronal loss and neuron synapse dysfunction (19). This finding is compatible with the known health effects associated with brevetoxins which have shown a neurotoxic effect in previous studies (20).

**Interleukin 6 (IL6)** encodes proteins involved in acute phase response, inflammation, and autoimmune response (21). This is compatible with previous studies, which showed that brevetoxins have immunosuppressant properties (22). Past research has shown that manatees experienced immune impairment following red tide exposures and this immune impairment places them at a greater risk of developing severe red tide illness (22-24).

**The zinc finger protein 804B (ZNF804B)** gene is involved in neuronal chemokine and cytokine regulation, autoimmune response, and immune activation (25).

The function of the encoded protein for the gene **Family with sequence similarity 186, member A (FAM186A)** is currently not known.

The **ankyrin 2, neuronal, transcript variant 1 (ANK2)** gene is associated with the stability of neuron synapses and is expressed in neuronal regions (26). In a study of neuromuscular junction (NMJs), ANK2 was recognized as one of the mutated genes identified in areas of instability (26). Lack of the ANK gene is related to synaptic microtubule dysfunction (26).

The **multimerin 1 (MMRN1)** gene is associated with bleeding disorders. It is expressed in endothelium and platelets (27). The down-regulated gene expression of MMRN1 is compatible with the potential hemolytic effect of brevetoxins, which has led to hemorrhaging in manatees during past red tide episodes (22, 28).

The **XK, Kell blood group complex subunit-related family, member 6 (XKR6)** gene is thought to have a role in immune function. The XKR6 gene was down-regulated in persons with eosinophilic esophagitis (EoE), a type of tissue-specific severe allergic sensitivity to certain foods (29). EoE produces an allergic reaction, which includes inflammation and causes the esophagus to swell. This condition is often seen in humans with allergies, eczema, and asthma (29).

The **cysteine-rich, angiogenic inducer, 61 (CYR61)** promotes endothelial cells adhesion. It’s down-regulation was associated with leiomyoma tumors or smooth muscle neoplasms and nonsmall cell lung cancers in previous studies (30).

**Forkhead box A1 (FOXA1)** gene deficiencies are associated with a disruption of albumin and transthyretin liver markers (31). In this study, transthyretin was down-regulated and confirmed through Western blot.

References:

1. Kim N, Takami M, Rho J, Josien R, Choi Y. A novel member of the leukocyte receptor complex regulates osteoclast differentiation. The Journal of experimental medicine. 2002;195(2):201-9.

2. Merck E, Gaillard C, Gorman DM, Montero-Julian F, Durand I, Zurawski SM, et al. OSCAR is an FcRgamma-associated receptor that is expressed by myeloid cells and is involved in antigen presentation and activation of human dendritic cells. Blood. 2004;104(5):1386-95.

3. Xu L, Kelvin DJ, Ye GQ, Taub DD, Ben-Baruch A, Oppenheim JJ, et al. Modulation of IL-8 receptor expression on purified human T lymphocytes is associated with changed chemotactic responses to IL-8. J Leukoc Biol. 1995;57(2):335-42.

4. Fu G, Rybakin V, Brzostek J, Paster W, Acuto O, Gascoigne NR. Fine-tuning T cell receptor signaling to control T cell development. Trends in immunology. 2014;35(7):311-8.

5. Peirce MJ, Brook M, Morrice N, Snelgrove R, Begum S, Lanfrancotti A, et al. Themis2/ICB1 is a signaling scaffold that selectively regulates macrophage Toll-like receptor signaling and cytokine production. PloS one. 2010;5(7):e11465.

6. Corry DB, Kiss A, Song LZ, Song L, Xu J, Lee SH, et al. Overlapping and independent contributions of MMP2 and MMP9 to lung allergic inflammatory cell egression through decreased CC chemokines. FASEB journal : official publication of the Federation of American Societies for Experimental Biology. 2004;18(9):995-7.

7. Tschesche H, Knäuper V, Krämer S, Michaelis J, Oberhoff R, Reinke H. Latent collagenase and gelatinase from human neutrophils and their activation. Matrix (Stuttgart, Germany). 1991;Supplement 1:245-55.

8. Joehanes R, Ying S, Huan T, Johnson AD, Raghavachari N, Wang R, et al. Gene expression signatures of coronary heart disease. Arterioscler Thromb Vasc Biol. 2013;33(6):1418-26.

9. Berger P, Bonneick S, Willi S, Wymann M, Suter U. Loss of phosphatase activity in myotubularin-related protein 2 is associated with Charcot-Marie-Tooth disease type 4B1. Human molecular genetics. 2002;11(13):1569-79.

10. Previtali SC, Zerega B, Sherman DL, Brophy PJ, Dina G, King RH, et al. Myotubularin-related 2 protein phosphatase and neurofilament light chain protein, both mutated in CMT neuropathies, interact in peripheral nerve. Human molecular genetics. 2003;12(14):1713-23.

11. Trainer VL, Baden DG. High affinity binding of red tide neurotoxins to marine mammal brain. Aquatic Toxicology. 1999;46(2):139-48.

12. Dandoy-Dron F, Griffond B, Mishal Z, Tovey MG, Dron M. Scrg1, a novel protein of the CNS is targeted to the large dense-core vesicles in neuronal cells. Eur J Neurosci. 2003;18(9):2449-59.

13. Wicher KB, Fries E. Prohaptoglobin is proteolytically cleaved in the endoplasmic reticulum by the complement C1r-like protein. Proceedings of the National Academy of Sciences of the United States of America. 2004;101(40):14390-5.

14. Wicher KB, Fries E. Haptoglobin, a hemoglobin-binding plasma protein, is present in bony fish and mammals but not in frog and chicken. Proceedings of the National Academy of Sciences of the United States of America. 2006;103(11):4168-73.

15. Langlois MR, Delanghe JR. Biological and clinical significance of haptoglobin polymorphism in humans. Clinical chemistry. 1996;42(10):1589-600.

16. Lazensky R, Walsh M, Chen S, Hunter M, Larkin I, Silva-Sanchez C, et al. Investigating an Increase in Florida Manatee (Trichechus Manatus Latirostris) Mortalities in 2013 Using a Proteomic Analysis. In preparation. 2019.

17. Gulhar R, Jialal I. Physiology, Acute Phase Reactants. StatPearls. Treasure Island (FL)2019.

18. Fenster SD, Garner CC. Gene structure and genetic localization of the PCLO gene encoding the presynaptic active zone protein Piccolo. International journal of developmental neuroscience : the official journal of the International Society for Developmental Neuroscience. 2002;20(3-5):161-71.

19. Ahmed MY, Chioza BA, Rajab A, Schmitz-Abe K, Al-Khayat A, Al-Turki S, et al. Loss of PCLO function underlies pontocerebellar hypoplasia type III. Neurology. 2015;84(17):1745-50.

20. Kirkpatrick B, Fleming LE, Squicciarini D, Backer LC, Clark R, Abraham W, et al. Literature Review of Florida Red Tide: Implications for Human Health Effects. Harmful Algae. 2004;3(2):99-115.

21. Kopf M, Baumann H, Freer G, Freudenberg M, Lamers M, Kishimoto T, et al. Impaired immune and acute-phase responses in interleukin-6-deficient mice. Nature. 1994;368(6469):339-42.

22. Bossart GD, Baden DG, Ewing RY, Roberts B, Wright SD. Brevetoxicosis in manatees (Trichechus manatus latirostris) from the 1996 epizootic: gross, histologic, and immunohistochemical features. Toxicologic pathology. 1998;26(2):276-82.

23. Walsh CJ, Butawan M, Yordy J, Ball R, Flewelling L, de Wit M, et al. Sublethal red tide toxin exposure in free-ranging manatees (Trichechus manatus) affects the immune system through reduced lymphocyte proliferation responses, inflammation, and oxidative stress. Aquat Toxicol. 2015;161:73-84.

24. Walsh CJ, Luer CA, Noyes DR. Effects of environmental stressors on lymphocyte proliferation in Florida manatees, Trichechus manatus latirostris. Veterinary immunology and immunopathology. 2005;103(3-4):247-56.

25. Chen J, Lin M, Hrabovsky A, Pedrosa E, Dean J, Jain S, et al. ZNF804A Transcriptional Networks in Differentiating Neurons Derived from Induced Pluripotent Stem Cells of Human Origin. PloS one. 2015;10(4):e0124597.

26. Koch I, Schwarz H, Beuchle D, Goellner B, Langegger M, Aberle H. Drosophila ankyrin 2 is required for synaptic stability. Neuron. 2008;58(2):210-22.

27. Hayward CP, Cramer EM, Song Z, Zheng S, Fung R, Masse JM, et al. Studies of multimerin in human endothelial cells. Blood. 1998;91(4):1304-17.

28. Jessup DA, Miller MA, Ryan JP, Nevins HM, Kerkering HA, Mekebri A, et al. Mass stranding of marine birds caused by a surfactant-producing red tide. PloS one. 2009;4(2):e4550.

29. Kottyan LC, Davis BP, Sherrill JD, Liu K, Rochman M, Kaufman K, et al. Genome-wide association analysis of eosinophilic esophagitis provides insight into the tissue specificity of this allergic disease. Nat Genet. 2014;46(8):895-900.

30. Sampath D, Zhu Y, Winneker RC, Zhang Z. Aberrant expression of Cyr61, a member of the CCN (CTGF/Cyr61/Cef10/NOVH) family, and dysregulation by 17 beta-estradiol and basic fibroblast growth factor in human uterine leiomyomas. The Journal of clinical endocrinology and metabolism. 2001;86(4):1707-15.

31. Lee CS, Friedman JR, Fulmer JT, Kaestner KH. The initiation of liver development is dependent on Foxa transcription factors. Nature. 2005;435(7044):944-7.
