# Supplementary material for: Investigating the gene expression profiles of rehabilitated Florida manatees (Trichechus manatus latirostris) following red tide exposure
Source: PLoS One. 2020 Jul 2;15(7):e0234150. doi: 10.1371/journal.pone.0234150 (PMC7331979; doi:10.1371/journal.pone.0234150)
Supplement: S2 File — (DOCX) [file pone.0234150.s006.docx]

Symbols:

| Upregulated (Red) | | Downregulated (Blue) | | |
| --- | --- | --- | --- | --- |
| ANXA3 | annexin A3 | ANK2 | ankyrin 2, neuronal |  |
| ARMC9 | armadillo repeat containing 9 | BIRC3 | baculoviral IAP repeat containing 3 |  |
| BST1 | bone marrow stromal cell antigen 1 | CCR5 | chemokine (C-C motif) receptor 5 |  |
| CA12 | carbonic anhydrase XII | CD69 | CD69 molecule |  |
| CARD6 | caspase recruitment domain family, 6 | CD80 | CD80 molecule |  |
| CASP3 | caspase 3, apoptosis-related cys peptidase | CD83 | CD83 molecule |  |
| CCR2 | chemokine (C-C motif) receptor 2 | CDK7 | cyclin-dependent kinase 7 |  |
| CCR8 | chemokine (C-C motif) receptor 8 | CSPG4 | chondroitin sulfate proteoglycan 4 |  |
| CD300LF | CD300 molecule-like family member f | CTLA4 | cytotoxic T-lymphocyte-associated protein 4 |  |
| CX3CR1 | chemokine (C-X3-C motif) receptor 1 | CXCL5 | chemokine (C-X-C motif) ligand 5 |  |
| CXCR1 | chemokine (C-X-C motif) receptor 1 | CXCR4 | chemokine (C-X-C motif) receptor 4 |  |
| CXCR2 | chemokine (C-X-C motif) receptor 2 | DUSP10 | dual specificity phosphatase 10 |  |
| FCGR3A | Fc fragment of IgG, low affinity IIIa, CD16a | DUSP4 | dual specificity phosphatase 4 |  |
| HERC5 | hect domain and RLD 5 | EGR1 | early growth response 1 |  |
| HP | haptoglobin | F3 | coagulation factor III (thromboplasti) |  |
| IFIH1 | interferon induced with helicase C1 | FLG | filaggrin |  |
| IL18RAP | interleukin 18 receptor accessory protein | FOS | FBJ murine osteosarcoma viral oncogene |  |
| ITGA4 | integrin, alpha 4 (antigen CD49D, alpha 4) | FST | follistatin |  |
| KLHL24 | kelch-like 24 (Drosophila) | GAD2 | glutamate decarboxylase 2 |  |
| LCN2 | lipocalin 2 | GADD45A | growth arrest & DNA-damage-inducible, a |  |
| LTB | lymphotoxin beta (TNF superfamily3) | GADD45G | growth arrest & DNA-damage-inducible, g |  |
| MAPK14 | mitogen-activated protein kinase 14 | GCH1 | GTP cyclohydrolase 1 |  |
| MMP9 | matrix metallopeptidase 9 | GCNT2 | glucosaminyl (N-acetyl) transferase 2, |  |
| NAIP | NLR family, apoptosis inhibitory protein | GMFB | glia maturation factor, beta |  |
| NLRC4 | NLR family, CARD domain containing 4 | HLA-B | major histocompatibility complex, I, B |  |
| OSCAR | osteoclast associated, immunoglobulin Recep | HLA-C | major histocompatibility complex, I, C |  |
| PDCD1LG2 | programmed cell death 1 ligand 2 | HNRNPA1 | heterogeneous nuclear ribonucleoprotein A1 |  |
| PGD | phosphogluconate dehydrogenase | HSPA14 | heat shock 70kDa protein 14 |  |
| PTPN6 | protein tyrosine phosphatase, non-receptor 6 | HSPA5 | heat shock 70kDa protein 5 (78kDa) |  |
| RNASEL | ribonuclease L (synthetase-dependent) | ICAM1 | intercellular adhesion molecule 1 |  |
| S100A8 | S100 calcium binding protein A8 | ICOSLG | inducible T-cell co-stimulator ligand |  |
| S100A9 | S100 calcium binding protein A9 | IL10 | interleukin 10 |  |
| SERPINB9 | serpin peptidase inhibitor, clade B, 9 | IL1A | interleukin 1, alpha |  |
| SH2D1B | SH2 domain containing 1B | IL1B | interleukin 1, beta |  |
| TLR7 | toll-like receptor 7 | IL6 | interleukin 6 (interferon, beta 2) |  |
| TLR8 | toll-like receptor 8 | ILF3 | interleukin enhancer binding factor 3, 90kDa |  |
|  |  | INHBA | inhibin, beta A |  |
|  |  | INSIG1 | insulin induced gene 1 |  |
|  |  | IRG1 | immunoresponsive 1 homolog (mouse) |  |
|  |  | JUN | jun proto-oncogene |  |
|  |  | JUNB | jun B proto-oncogene |  |
|  |  | LAX1 | lymphocyte transmembrane adaptor 1 |  |
|  |  | MAP3K8 | mitogen-activated prot kinase kinase kinase 8 |  |
|  |  | MDM2 | Mdm2 p53 binding protein homolog (mouse) |  |
|  |  | MOAP1 | modulator of apoptosis 1 |  |
|  |  | NFKBIA | nuclear factor of kappa light polypeptide gene enhancer in B-cells inhibitor, alpha |  |
|  |  | NFKBIZ | nuclear factor of kappa light polypeptide gene enhancer in B-cells inhibitor, zeta |  |
|  |  | ODC1 | ornithine decarboxylase 1 |  |
|  |  | PIGA | phosphatidylinositol glycan anchor, A |  |
|  |  | PNRC1 | proline-rich nuclear receptor coactivator 1 |  |
|  |  | PTGER4 | prostaglandin E receptor 4 (subtype EP4) |  |
|  |  | PTGS2 | prostaglandin-endoperoxide synthase 2 |  |
|  |  | RGS1 | regulator of G-protein signaling 1 |  |
|  |  | SIRT1 | sirtuin 1 |  |
|  |  | SKIL | SKI-like oncogene |  |
|  |  | SLC25A33 | solute carrier family 25, member 33 |  |
|  |  | THBS1 | thrombospondin 1 |  |
|  |  | TLR4 | toll-like receptor 4 |  |
|  |  | TNFSF4 | tumor necrosis factor superfamily, 4 |  |
|  |  | TRAF6 | TNF receptor-associated factor 6 |  |
|  |  | XBP1 | X-box binding protein 1 |  |
|  |  | ZBTB16 | zinc finger and BTB domain containing 16 |  |
